# An Efficient Rank Based Approach for Closest String and Closest Substring

**DOI:** 10.1371/journal.pone.0037576

**Published:** 2012-06-04

**Authors:** Liviu P. Dinu, Radu Ionescu

**Affiliations:** Faculty of Mathematics and Computer Science, University of Bucharest, Bucharest, Romania; National Taiwan University, Taiwan

## Abstract

This paper aims to present a new genetic approach that uses rank distance for solving two known NP-hard problems, and to compare rank distance with other distance measures for strings. The two NP-hard problems we are trying to solve are closest string and closest substring. For each problem we build a genetic algorithm and we describe the genetic operations involved. Both genetic algorithms use a fitness function based on rank distance. We compare our algorithms with other genetic algorithms that use different distance measures, such as Hamming distance or Levenshtein distance, on real DNA sequences. Our experiments show that the genetic algorithms based on rank distance have the best results.

## Introduction

### Motivation

In many important problems in computational biology a common task is to compare a new DNA sequence with sequences that are already well studied and annotated. Sequences that are similar would probably have the same function, or, if two sequences from different organisms are similar, there may be a common ancestor sequence [1]. Another important problem with practical motivations for biologists is related to the finding of motifs or common patterns in a set of given DNA sequences. A typical case where the last mentioned problem occurs is, for example, when one needs to design genetic drugs with structure similar to a set of existing sequences of RNA [2]. Other applications in computational biology which involve this task are (from a rich literature): PCR primer design [3], [2], genetic probe design [2], antisense drug design [4], finding unbiased consensus of a protein family [5], motif finding [6], [7] etc. In many situations all these applications share a task that requires the design of a new DNA or protein sequence that is very similar to (a substring of) each of the given sequences.

In computational biology the problem that deals with this task is known as the closest string problem (CSP): given a set 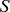 of strings over an alphabet 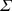, find the string which is the most similar to the strings from 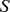. The similarity measure varies according to the application. The CSP was studied first time in the area of coding theory, to determine the best encoding of a set of messages [8], and the measure used to compare the strings was the Hamming distance.

The standard method used in computational biology for sequence comparison is by sequence alignment. Sequence alignment is the procedure of comparing two sequences (pairwise alignment) or more sequences (multiple alignment) by searching for a series of individual characters or characters patterns that are in the same order in the sequences. Algorithmically, the standard pairwise alignment method is based on dynamic programming; the method compares every pair of characters of the two sequences and generates an alignment and a score, which is dependent on the scoring scheme used, i.e. a scoring matrix for the different base-pair combinations, match and mismatch scores, or a scheme for insertion or deletion (gap) penalties.

Although dynamic programming for sequence alignment is mathematically optimal, it is far too slow for comparing a large number of bases, and too slow to be performed in a reasonable time.

Also, since some of the search solutions are inaccurate from a biological point of view, alternative approaches periodically are explored in computational biology. This important problem, known also as DNA sequence comparison, is ranked in the top of two lists with major open problems in bioinformatics [9], [10].

The standard distances with respect to the alignment principle are edit (Levenshtein) distance [11] or its ad-hoc variants. The study of rearrangement genome [12] was investigated also under Kendall tau distance (the minimum number of swaps needed to transform a permutation into the other).

To measure the similarity between strings Dinu proposes a new distance measure, termed *rank distance (RD)* [13], with applications in biology [14], natural language processing [15], authorship atribution [16]. Rank distance can be computed fast and benefits from some features of the edit distance.

To measure the distance between two strings with RD we scan (from left to right) both strings and for each letter from the first string we count the number of elements between its position in the first string and the position of its first occurrence in the second string. Finally, we sum up all these scores and obtain the rank distance. In other words, the rank distance measures the “gap” between the positions of a letter in the two given strings, and then sums up these values. Intuitively, the rank distance gives us the total non-alignment score between two sequences.

Clearly, the rank distance gives a score zero only to letters which are in the same position in both strings, as Hamming distance does (we recall that Hamming distance is the number of positions where two strings of the same length differ). On the other hand, an important aspect is the reduced sensitivity of the rank distance with respect to deletions and insertions. Reduced sensitivity is of paramount importance, since it allows the *ad hoc extension to arbitrary strings*, without affecting the low computational complexity. In contrast, the extensions of Hamming distance are mathematically optimal but computationally too heavy, and lead to the *edit-distance*, which is the base of the standard alignment principle. Thus, the rank distance sides with Hamming distance rather than Levenshtein distance as far as computational complexity is concerned: a significant indicator is the fact that in the Hamming and rank distance case the median string problem is tractable [17], while in the edit distance case it is NP-hard.

RD is easy to implement, does not use the standard alignment principle, and has an extremely good computational behavior. Another advantage of RD is that it imposes minimal hardware demands: it runs in optimal conditions on modest computers, reducing the costs and increasing the number of possible users. For example, the time needed to compare a DNA string of 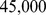 nucleotides length with other 150 DNA strings (with similar length), by using an laptop with 224 MB RAM and 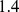 GHz processor is no more than six seconds.

Traditionally, the Closest String Problem (CSP) is related to Hamming distance and it tries to find a minimal integer 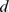 (and a corresponding string 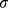 of length 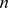) such that the maximal Hamming distance to any string in 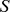 is at most 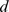. It all started from a code theory application [18]. There are recent studies that investigate CSP under Hamming distance with advanced programming techniques such as integer linear programming (ILP) [19].

In [18] it is shown that the decision problem associated with the Covering Radius of arbitrary binary codes is NP-complete. The Radius of a binary code 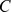 is the smallest integer 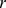 such that 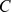 is contained in a radius-r ball of the Hamming metric space 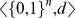. Starting from the problems of equivalence between computing the Radius and the Covering Radius problem [20], in [18] it is shown that the 3SAT problem is polynomially reducible to the Radius decision problem. There are a number of approximation algorithms and heuristics (e.g. [2], [7], [21]).

When CSP emerged in bioinformatics, the problem was investigated from many points of view. These investigations implied the use of different distances. The most intensive studied approach was the one based on edit distance. In [22], it is shown that closest string and median string (via edit distance) are NP-hard for alphabets of size at least 4 and for unbounded alphabets, respectively.

In many practical situations the alphabet is of fixed constant size (in computational biology, the DNA and protein alphabets are respectively of size 4 and 20). For some applications, one needs to encode the DNA or protein sequences on a binary alphabet that expresses only a binary property of the molecule, e.g. hydrophoby (for instance, this is the case in some protocols that identify similar DNA sequences [23]). In [24]–[25] it is shown that closest string and median string are NP-hard for finite and even binary alphabets. The existence of fast exact algorithms, when the number of input strings is fixed, is investigated in [24].

The study of genome rearrangement specific problems lead to the development of new problems related to closest string via various distances used in the investigations of this problems. Recently, in [26] it is shown that the CSP via swap distance (or Kendall distance) and CSP via element duplication distance (the element duplication distance between 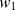 and 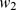 is the minimum number of element duplications needed to transform a string 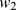 into a string 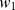) remain NP-hard too.

In [27] it is shown that the CSP and CSSP (*closest substring problem*) via rank distance are NP-hard. In this paper we use an approach based on genetic algorithms to propose an approximation of CSP and CSSP via rank distance.

### Preliminaries

In this section we introduce notation and mathematical preliminaries. We first introduce the rank distance and then we define closest string and closest substring problems.

A ranking is an ordered list and is the result of applying an ordering criterion to a set of objects. Formally,

**Definition 1.** Let 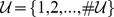 be a finite set of objects, named universe (we write 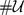 for the cardinality of 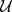). A ranking over 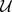 is an ordered list: 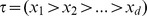, where 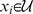 for all 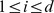, 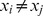 for all 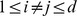, and 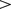 is a strict ordering relation on the set 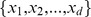.

A ranking defines a partial function on 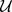 where for each object 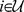, 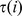 represents the position of the object 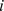 in the ranking 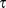. Observe that the objects with high rank in 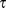 have the lowest positions.

The rankings that contain all the objects of an universe 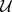 are termed *full rankings*, while the others are *partial rankings*. We define the order of an object 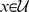 in a ranking 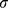 of length 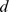, by 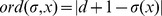. By convention, if 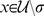, we have 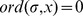.

**Definition 2.** Given two partial rankings 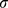 and 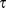 over the same universe 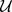, we define the rank distance between them as:
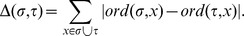



In [13] Dinu proves that 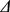 is a distance function. The rank distance is an extension of the Spearman footrule distance [28], defined below.

**Definition 3.** If 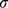 and 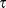 are two permutations of the same length, then 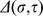 is named the Spearman footrule distance.

The rank distance is naturally extended to strings. The following observation is immediate: if a string does not contain identical symbols, it can be transformed directly into a ranking (the rank of each symbol is its position in the string). Conversely, each ranking can be viewed as a string, over an alphabet equal to the universe of the objects in the ranking. The next definition formalizes the transformation of strings that have identical symbols into rankings.

**Definition 4.** Let 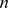 be an integer and let 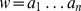 be a finite word of length 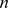 over an alphabet 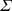. We define the extension to rankings of 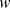, 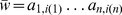, where 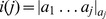 for all 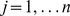 (i.e. the number of occurrences of 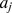 in the string 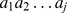).

**Example 1.** If 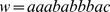 then
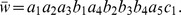



Observe that given 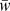 we can obtain 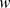 by simply deleting all the indexes. Note that the transformation of a string into a ranking can be done in linear time (by memorizing for each symbol, in an array, how many times it appears in the string [14]). We extend the rank distance to arbitrary strings as follows:

**Definition 5.** Given 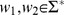, we define 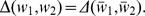.

**Example 2.** Consider the following two strings 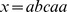 and 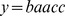. Then, 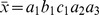 and 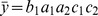. Thus, the rank distance between 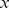 and 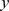 is the sum of the absolute differences between the orders of the characters in 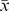 and 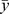.
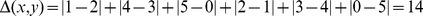



The computation of the RD between two rankings can be done in linear time in the cardinality of the universe. Our universe has precisely 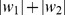 objects and, thus, the RD between 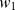 and 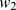 can be computed in linear time.

Let 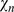 be the space of all strings of size 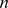 over an alphabet 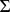 and let 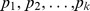 be 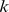 strings from 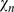. The center string problem is to find the center of the sphere of minimum radius that includes all the 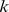 strings. An alternative formulation of the problem is to find a string from 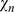 which minimizes the distance to all the input strings. We study the closest string problem under a metric defined by the rank distance. In our experiments, we compare rank distance with other metrics defined by Hamming distance and Levenshtein distance.

**Problem 1 (Closest string via rank distance).** Let 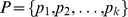 be a set of 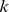 length 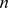 strings over an alphabet 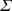. The closest string problem via rank distance (CSRD) is to find a minimal integer 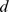 (and a corresponding string 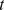 of length 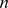) such that the maximum rank distance from 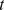 to any string in 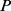 is at most 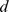. We say that 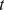 is the closest string to 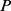 and we name 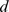 the radius. Formally, the goal is to compute:
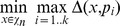



The CSSP is a generalization of CSP where the objective is to find a string similar to substrings of the input.

**Problem 2 (Closest substring via rank distance).** Let 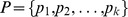 be a set of 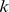 length 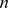 strings over an alphabet 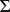. The closest substring problem via rank distance is to find a minimal integer 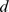 (and a corresponding string 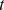 of length 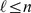) and a set 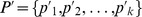, where 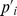 is a substring 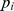 for all 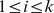 such that the maximum rank distance from 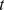 to any string in 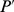 is at most 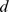. We say that 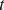 is the closest substring to 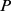 and we name 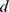 the radius. Formally, the goal is to compute:
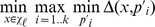



## Results and Discussion

### Experiments Design

We test the genetic algorithm using mitochondrial DNA sequences extracted from several mammals available in the EMBL database: human (*Homo sapiens*, V00662), common chimpanzee (*Pan troglodytes*, D38116), gorilla (*Gorilla gorilla*, D38114), donkey (*Equus asinus*, X97337), rat (*Rattus norvegicus*, X14848), mouse (*Mus musculus*, V00711), fat dormouse (*Myoxus glis*, AJ001562), and cow (*Bos taurus*, V00654). Mitochondrial DNA (mtDNA) is the DNA located in organelles called mitochondria. The DNA sequence of mtDNA has been determined from a large number of organisms and individuals, and the comparison of those DNA sequences represents a mainstay of phylogenetics, in that it allows biologists to elucidate the evolutionary relationships among species. In mammals, each double-stranded circular mtDNA molecule consists of 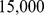
–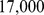 base pairs.

**Figure 1:**
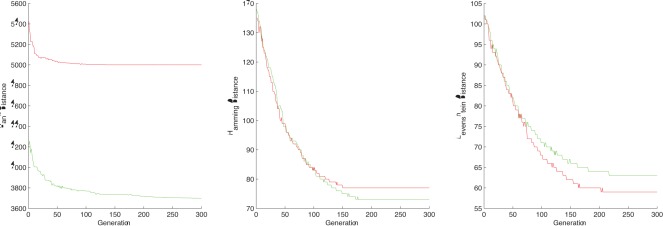
The distance evolution of the best chromosome at each step for - TEST CASE 1. GREEN  =  human-chimpanzee distance, RED  =  human-donkey distance.

In our experiments each mammal is represented by a single mtDNA sequence that comes from a single individual. We mention that DNA from two individuals of the same species differs by only 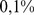. This means, for example, that mtDNA from two different humans differs by less than 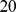 base pairs. Because this difference cannot affect our study, we conduct the experiments using a single mtDNA sequence for each mammal.

**Figure 2:**
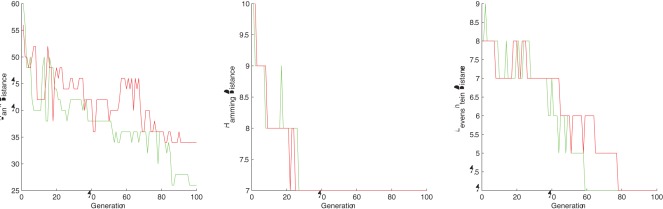
The distance evolution of the best chromosome at each step for - TEST CASE 2. GREEN  =  human-chimpanzee distance, RED  =  human-donkey distance.

For each of the two problems (CSP and CSSP) we design two similar experiments. We have another artificial experiment for CSSP, and another experiment for CSP with great interest for biologist.

**Figure 3:**
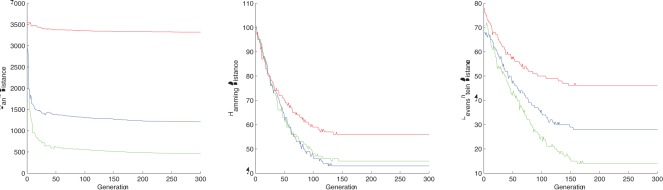
The distance evolution of the best chromosome at each step for - TEST CASE 3. GREEN  =  rat-house mouse distance, BLUE  =  rat-fat dormouse, RED  =  rat-cow distance.

For the first experiment we use the human, chimpanzee and donkey genomes. We want to find the closest string (or substring) of nucleotides between the human and chimpanzee DNAs on one hand, and between the human and donkey DNAs on the other hand. The goal of this experiment is to compare the distances obtained for the two strings (or substrings). Note that the donkey belongs to the Perissodactylae branch, while the human and the chimpanzee belong to the Primates branch. Since the human and the chimpanzee are both primates, the human-chimpanzee distance should be smaller than the human-donkey distance. In other words, we expect the biological classification of mammals to be reflected in the DNA.

**Figure 4:**
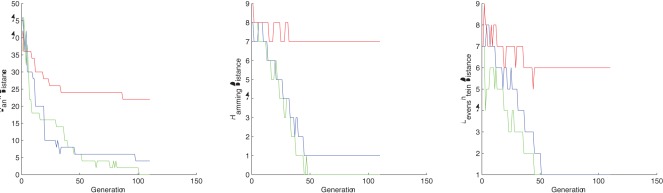
The distance evolution of the best chromosome at each step for - TEST CASE 4. GREEN  =  rat-house mouse distance, BLUE  =  rat-fat dormouse, RED  =  rat-cow distance.

For the second experiment we use the rat, house mouse, fat dormouse and cow genomes. As in the former case, we want to find the closest string (or substring) of nucleotides between the rat and house mouse DNAs, between the rat and fat dormouse DNAs, and between the rat and cow DNAs. The goal of this experiment is to compare the distances obtained for the three strings (or substrings). Note that the cow belongs to the Cetartiodactylae branch, while the rat, the house mouse, and the fat dormouse belong to the Rodentia branch. We expect the rat-house mouse distance and the rat-fat dormouse distance to be smaller than the rat-cow distance. We have chosen this experiment because in [14], where a clustering of genomes from 22 mammals is performed, the rat appears to be clustered near the cow and sheep rather than the house mouse and fat dormouse. This was contradictory to what we know from the biological classification of mammals. We wanted to see if our experiment can bring any arguments to support the well known fact from biology.

**Figure 5:**
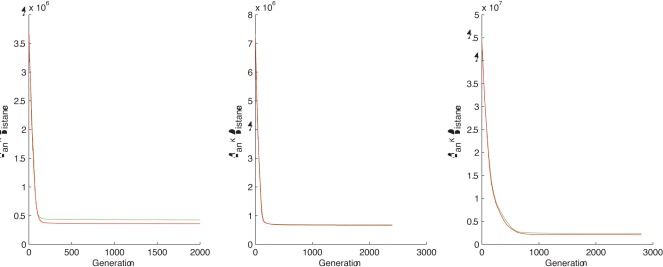
The distance evolution of the best chromosome at each step for - TEST CASE 7, 8 and 9, respectively. GREEN  =  human-chimpanzee distance, RED  =  human-gorilla distance.

We also use an artificial test case for the closest substring problem to point out our optimization of the genetic algorithm presented in [29] where we used the same artificial test case. We optimize our genetic algorithm to compute the rank distance measure in a linear time. We also use a hash table to store precomputed distances between DNA sequences.

**Table 1:** Chromosomes C1, C2 and C3.

C1	7	3	2	9	1	5	8	6	4
C2	4	8	1	6	3	7	9	5	2
C3	2	5	9	8	7	1	3	4	6

Note that another study that shows experiments using Hamming distance for CSP and CSSP is [19]. The authors present an ILP solution to solve these problems and they conclude that current ILP techniques are not really up to the task for the CSSP, except for small instances. Here we present an alternative approach (genetic algorithms) and we investigate it under different metrics.

**Table 2:** Recombined chromosomes C1 and C2 with same prefix.

C1	7	3	2	9	4	8	1	6	5
C2	4	8	1	6	7	3	2	9	5

Each of our experiments are performed using three different metrics: rank distance, Hamming distance and Levenshtein distance. We want to compare the results for each distance measure. We show graphs of the best candidate evolution for each metric used.

After we determine the metric that has the best results, we will perform another experiment (using only this metric) with great interest for biologists. At present, no definitive agreement on either the correct branching order or differential rates of evolution among the higher primates exists, despite the research in this area. Joining human with chimpanzee and the gorilla with the orangutan is currently favoured, but the alternatives that group humans with either gorillas or the orangutan rather than with chimpanzees also have support [30]. In our latest experiment we try to find out if our genetic algorithm solution can lead to one of these phylogenetic trees.

**Table 3:** Chromosomes C1, C2 and C3 with mutations.

C1	7	5	2	9	1	3	8	6	4
C2	4	8	1	6	3	7	9	5	2
C3	2	1	9	8	4	5	3	7	6

With two experiments and three distance measures for the closest string problem, we have six test cases with associated graphs. For the closest substring there is an extra artificial experiment, generating nine test cases and six graphs associated to the real DNA experiments. In our latest experiment we use the distance measure that has the best performance on the former test cases. We investigate only the closest strings for DNA sequences of variable lengths and we present three more graphs.

**Figure 6:**
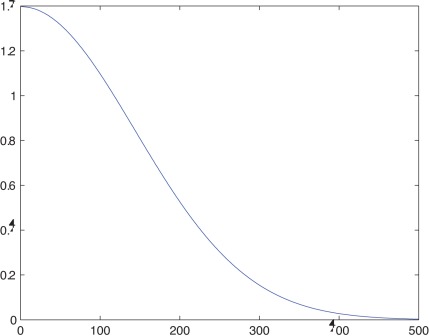
The graph of density probability function.

### Experiments Organisation

For each experiment we give the input strings, then we present the results obtained by using rank distance, Hamming distance and Levenshtein distance, respectively. An input string is a DNA sequence. The algorithm designed for CSRD needs at least two DNAs (of same length) to produce an output DNA sequence. The output DNA is the closest string to the input strings computed with rank distance. Using Hamming or Levenshtein distance in the selection process of the genetic algorithm is analogous. The algorithm designed for CSSRD need two DNAs (not necessary of same length) to produce the output DNA that represents the closest substring.

Let us describe the genetic algorithm parameters and the format of the input and output data. The population size represents the number of chromosomes in a single generation. The crossover probability represents the percent of chromosomes (from a single generation) that get involved in the crossover operation. The mutation probability is similar to the crossover probability only that the chromosomes are mutated. The number of strings (DNA sequences) gives the number of input strings. The size of each DNA sequence is the number of nucleotides in every DNA sequence. We use different input parameters for each problem that we are trying to solve. The input parameters used for one experiment are the same for every investigated metric. We want to compare only the metrics used, without changing the genetic algorithm parameters or the genetic operations involved.

The average time represents the mean time for 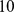 runs on the same input data using a computer with Intel Core i5 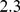 GHz processor and 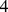 GB of RAM memory. The distance achieved for each test case is the same or less for 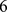 out of 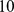 runs.

### Human-Chimpanzee-Donkey Experiment

There are two different settings for this experiment corresponding to CSP and CSSP, respectively. We present the test cases and results separately for each setting.

#### CSP setting

In this setting we use the first 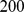 nucleotides extracted from each of the human, chimpanzee and donkey DNA sequences. By convention, single strands of DNA and RNA sequences are written in 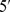-to-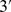 direction. When we talk about "the first nucleotides" in a DNA sequence through this paper we understand the nucleotides that are closest to the 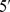 end. We want to determine the human-chimpanzee and human-donkey closest strings which also have 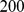 nucleotides.

1. RANK DISTANCE TEST CASE 1: Population size: 2500; number of generations: 300; crossover probability: 0.36; mutation probability: 0.002; size of each DNA sequence: 200.

**HUMAN-CHIMPANZEE RESULT:** Average time: 22 seconds; Distance achieved: 3698; Closest string: G T A C T A C G C G T T T A C T C T A C C A A A C G C A T A C T G A C A A A T G T C T G T T A G A T G G A T C C A T C T C C G C G T G T A C T G T C T A A A A G C G T A G C G T C A C G T A C G T C A A G C A G T G T T T C A G T C C C A C A A T C C A T T G C A C A T T A C T G A G C T C T C C A T T C G T C T C A C T C T T T T T A C G A A C A A T A T T A T C A A T G C A A A C G T G G G C C C T C T T C.

**HUMAN-DONKEY RESULT:** Average time: 22 seconds; Distance achieved: 5001; Closest string: T G A A G A G C A T T C C A T A T C T A A C T C C T G A A G T A C A C G A A C G G A T A T G C A C T T T G C T T C G T T A C A C T A G C G T G G A C G T A C A T T C T C G G C T G A C C T T G G G C A T A T A A T A T T A A A G T A A C G G A G T C T A C A T C T A A T A T C A T C G T A A C C C A T A G A A T G T T A T A C C C T C A T C G T C C T T C G C C C A A G T G C C C T G C T T A A C T T C T C A T.

2. HAMMING DISTANCE TEST CASE 1: Population size: 2500; number of generations: 300; crossover probability: 0.36; mutation probability: 0.002; size of each DNA sequence: 200.

**HUMAN-CHIMPANZEE RESULT:** Average time: 1 min 14 seconds; Distance achieved: 73; Closest string: G A T C A T G T G G C T A T C A C C C T C A A A G C C A C T C A C G G G A A C T G T T C A G A C A T T T T T A C A T T A C C C C A T G A A G A T A T G C G C G T G G T A C T A T T C T G T C A A G C A G C A G T C A G A A A A C T C A C T C T T G C A A T A A C T G T C T T T G C T T G C T T C A T C A T C T T A A T C T C T A T C A T C A C T A G G T A C A A T A A T A C A G G C C G A C G C A C T G C A G C.

**HUMAN-DONKEY RESULT:** Average time: 1 min 13 seconds; Distance achieved: 77; Closest string: G A T C A C A G A G C T A A A A G A C A A C A A A C C A C G C A C C T G A A A A T G C C A A G A T T T T G G T T C C T A C G C C T T G G G C A T A T A C A C T C G A T C C C G T T C T G A T A C T C T G T A A C C G G T G C A A C C A C T C A T G C A A G A T T C G T C A T T C C T G C C T G A A T G A T C T C T T T A T T G A A C T C T C C G A T C T T A A G G A G C A G G T G C G A A C A A A A T T A C T A.

3. LEVENSHTEIN DISTANCE TEST CASE 1: Population size: 2500; number of generations: 300; crossover probability: 0.36; mutation probability: 0.002; size of each DNA sequence: 200.

**HUMAN-CHIMPANZEE RESULT:** Average time: 24 min 12 seconds; Distance achieved: 63; Closest string: G T A T A C A C A G C T C T A C C C C C T A A A G C A A T A C C A C G G A A G A T C T T C C A T G G A T T T A T A T C A T C C T C T A A G C A A C A T G C A T G G T A G C C T T G C G A T T C G A T T G A G C T C G T G A G A C C C T A T A T C G C A T A C T G A T C C C C G A T C C T G G T C A T C C T A T T A A T C A T C C A T G T A A A G T T A C A A G T A T T A C A G C G C G C A G C A A T T A C A A C.

**HUMAN-DONKEY RESULT:** Average time: 24 min 11 seconds; Distance achieved: 59; Closest string: G T T C A A T G T A C T A T C A C G A T A T A A A T C A A G G A G C T G T C A A T G C A C T T G G T A G T T T C C T C T G C G C T A T G C A C A C A T A G G G C A T T G C G A C C T G G A G C C T T A T T A T T A C T A T G A A G C A G A T T A A C A T G C A T T G A T T C C T G C C T C C C C A T A T A A T C C T C T A A A T C G C A C T C T A G A T C A A A T T A C A G G C G A A C A A G A C T C T A C T A.

#### CSSP setting

In this setting we use the first 300 nucleotides extracted from the human, chimpanzee and donkey genomes. We want to determine the human-chimpanzee and human-donkey closest substrings of 24 nucleotides.

Here the substring size input parameter represents the desired length of the best substring (which represents the output of the genetic algorithm).

1. RANK DISTANCE TEST CASE 2: Population size: 500; number of generations: 100; crossover probability: 0.36; mutation probability: 0.02; size of each DNA sequence: 300; substring size: 24.

**HUMAN-CHIMPANZEE RESULT:** Average time: 39 seconds; Distance achieved: 26; Closest substring: C T T A G T A A C T A T A T C G A G A C A A G C.

**HUMAN-DONKEY RESULT:** Average time: 40 seconds; Distance achieved: 34; Closest substring: A C A T G C C T A T C T A C C C G T A A T A C C.

2. HAMMING DISTANCE TEST CASE 2: Population size: 500; number of generations: 100; crossover probability: 0.36; mutation probability: 0.02; size of each DNA sequence: 300; substring size: 24.

**HUMAN-CHIMPANZEE RESULT:** Average time: 1 min 36 seconds; Distance achieved: 7; Closest substring: C T A C A C A C G C A A G C C T T C C C T G C A.

**HUMAN-DONKEY RESULT:** Average time: 1 min 38 second; Distance achieved: 7; Closest substring: A C G T A C G A A C C A T A C T A C A A G C T A.

3. LEVENSHTEIN DISTANCE TEST CASE 2: Population size: 500; number of generations: 100; crossover probability: 0.36; mutation probability: 0.02; size of each DNA sequence: 300; substring size: 24.

**HUMAN-CHIMPANZEE RESULT:** Average time: 7 min 4 seconds; Distance achieved: 4; Closest substring: T T G A T T C C T G C C T A T C T A T T A G C T.

**HUMAN-DONKEY RESULT:** Average time: 7 min 3 seconds; Distance achieved: 4; Closest substring: A T G C T A C T C T T A A T C G C A C C T A C G.

#### Observations

First, we must point out that rank distance, Hamming distance and Levenshtein distance use different scales, i.e. a rank distance of 100 is not equivalent to a Hamming distance of 100, nor a Hamming distance of 100 to a Levenshtein distance of 100. We would also like to point out that rank distance has a finer scale, possibly being able to detect subtle differences between DNA strings.

As one might expect, the results indicate that the human genome is closer to the chimpanzee genome, than it is to the donkey genome.

In the CSP setting, rank distance shows a great difference between the human-chimpanzee closest string and the human-donkey closest string. Levenshtein distance indicates that humans are closer related to donkeys than to chimpanzees, while Hamming gives the expected result as rank distance does. Both Hamming and Levenshtein distances show small differences between the two analysed strings. The evolution of the best closest string candidate for each distance measure is given in Figure 1.

In the CSSP setting, RD is the only distance that can catch the subtle difference between the human-chimpanzee closest substring and the human-donkey closest substring, even if we use only 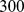 nucleotides. Hamming and Levenshtein distances are unable to make any difference between the two closest substrings. Figure 2 presents the graphs with the best closest substring candidate according to rank distance, Hamming distance and Levenshtein distance, respectively.

In both CSP and CSSP settings, rank distance clearly outperforms Hamming and Levenshtein distances (see Figures 1 and 2).

### Rat-Mouse-Cow Experiment

As for the Human-Chimpanzee-Donkey experiment, there are two different settings corresponding to CSP and CSSP. We present the test cases and results separately for each setting.

#### CSP setting

In this setting we use the first 150 nucleotides extracted from each of the rat, house mouse, fat dormouse and cow DNA sequences. We want to determine the rat-house mouse, rat-fat dormouse and rat-cow closest strings which also have 150 nucleotides.

1. RANK DISTANCE TEST CASE 3: Population size: 1800; number of generations: 300; crossover probability: 0.36; mutation probability: 0.005; size of each DNA sequence: 150.

**RAT-HOUSE MOUSE RESULT:** Average time: 12 seconds; Distance achieved: 454; Closest string: G T T G A A T C G T T A A T A T A C A A A G C A A G T A C A T G A A T C A G A A G T G A T A T T C T A A A A G C T T A G C A A C C A T C A A A T A T G T G G C C G T G T T C T A C A T T T A A G T G A A G A T G T A A A T C A A A C C T A A G C A T C A T G A C A T G C G A A T C A A G C A T A C C T A T T.

**RAT-FAT DORMOUSE RESULT:** Average time: 12 seconds; Distance achieved: 1209; Closest string: G T A T A C T G T A G T A T A A A A A A T C T G A G A C C A T G A T A A T G T A C A G T A G G A T A C A T A C C T A A C C G C A A C A A T T G A T G C C G T G T A C G C T T A A T T T C A A T G T C T C T A G C A G G A A G A A A A T T T G C A A A C T T C C A A C G A A A G T C G C T A A A T G T C C A T.

**RAT-COW RESULT:** Average time: 12 seconds; Distance achieved: 3321; Closest string: G T A T A A C A T G T C A C T G A A C C G A A T A C T A G T A A T G A A A A T T C G G C T C T T A T G C A A G A C T T A T A C T T T C A G G A G G A T C G A T T T T A G A A C A T G A A A A T G C T A G G C T G T A G T G G C G T A G A T C A C T A G G C A G C T G C T T G T T C T T T T G T C A A C T G G.

2. HAMMING DISTANCE TEST CASE 3: Population size: 1800; number of generations: 300; crossover probability: 0.36; mutation probability: 0.005; size of each DNA sequence: 150.

**RAT-HOUSE MOUSE RESULT:** Average time: 42 seconds; Distance achieved: 45; Closest string: G T T A A T G T A G C T T A T T A A C A A G G A A A G G A A T T G A A A A T G T T T A G T G G G T T C A A T A T T C C C A A T A A C C C A A A G G G T T G G T C C C G G G C C T G T A A A T A A A T T A A G G G T A G A A T A A A C A T T C A A A A C C C C C A A A A A C C G G G T T A A A A C C C T T T A.

**RAT-FAT DORMOUSE RESULT:** Average time: 41 seconds; Distance achieved: 43; Closest string: G T T A A T G T A G C T T A T A A T A A G C A A A A C C A T T A A A A A G C T T T G G A T G G A A T C T A A A A C C C C T A A A A C A A A A A G T T T G G G C C C A G G C T T T T T A A T T G T T T G T A G G A A A A A T A A A C A T T G C A A C A A T C A C G A C A C C G G T A T A A A A C C C T T T A C.

**RAT-COW RESULT:** Average time: 41 seconds; Distance achieved: 56; Closest string: A T T A A T G G A T A A T C T G C T A A T G C A A A G A C A T G A C A A T G C T G T G A T A G A T T T A G A A A T T C T A T A A T C A G G A A G G T T T T G G C A T T C A G C T A T G G T T G A C T G A G G G T A T G A T T C G A C A C A T A A A C T T C A A T A G G C C T T A G C A G A A T C T T T A G A.

3. LEVENSHTEIN DISTANCE TEST CASE 3: Population size: 1800; number of generations: 300; crossover probability: 0.36; mutation probability: 0.005; size of each DNA sequence: 150.

**RAT-HOUSE MOUSE RESULT:** Average time: 9 min 28 seconds; Distance achieved: 14; Closest string: G T T A A T G T A G C T T A T A A T A A A G C A A A G C A C T G A A A A G C T T A G A T G G A T C A A A T G A T C C C A T A A A C A C A A A G G T T T G G T C C T G G C C T A A A T A A T T A G A G G T A A A G A T C T A C A C A T G C A A A C C T C C A T A G A C C G G T G T A A A C A T C C C G T T A A.

**RAT-FAT DORMOUSE RESULT:** Average time: 9 min 29 seconds; Distance achieved: 28; Closest string: T T A A T G A G C T T A A A A G C A A A G C A A C T G A A A T G C T T A G A T G G T A G C A A A T A T C C C A T A A A C A C A A A G G T T C T G G T C C C A G C C T T C T A T T A A T T A G A T T G T A T A G C A A G A T T A C A C A T G C A A C A T C A T G A A C C T G G T G T A A G A A T C C C T T A A.

**RAT-COW RESULT:** Average time: 9 min 29 seconds; Distance achieved: 46; Closest string: G A C T A A T G G C T A T C A G A A T G C A A A G C A C A T G A A C A T G C T G C T G A G A T A G A T T T G A A A A T C T T T A A T A C T G G A A G G G T T G C T C C T G G A C T C A T A G C T A T G G A C G T A A G G C T T G A C A C A G C A T A C A T T G T A C C G G A G T A A A A T G C A C T T A A G.

#### CSSP setting

In this setting we use the first 300 nucleotides extracted from the rat, house mouse, fat dormouse and cow genomes. We want to determine the rat-house mouse, rat-fat dormouse and rat-cow closest substrings of 24 nucleotides.

The substring size parameter is the desired length of the best substring.

1. RANK DISTANCE TEST CASE 4: Population size: 700; number of generations: 110; crossover probability: 0.36; mutation probability: 0.03; size of each DNA sequence: 300; substring size: 24.

**RAT-HOUSE MOUSE RESULT:** Average time: 1 min 25 seconds; Distance achieved: 0; Closest substring: A A A G C A A A G C A C T G A A A A T G C T T A.

**RAT-FAT DORMOUSE RESULT:** Average time: 1 min 24 seconds; Distance achieved: 4; Closest substring: A T A A G A C A A G C A C T G A A A A T G C T T.

**RAT-COW RESULT:** Average time: 1 min 25 seconds; Distance achieved: 22; Closest substring: A G A T A C G T T C A G T A C A T G A G T A C C.

2. HAMMING DISTANCE TEST CASE 4: Population size: 600; number of generations: 110; crossover probability: 0.36; mutation probability: 0.03; size of each DNA sequence: 300; substring size: 24.

**RAT-HOUSE MOUSE RESULT:** Average time: 2 min 5 seconds; Distance achieved: 0; Closest substring: T C A G C A G T G A T A A A T A T T A A G C A A.

**RAT-FAT DORMOUSE RESULT:** Average time: 2 min 4 seconds; Distance achieved: 1; Closest substring: C C C C A T A A A C A C A A A G G T T T G G T C.

**RAT-COW RESULT:** Average time: 2 min 4 seconds; Distance achieved: 7; Closest substring: G T A A T T G G A C A T A A A T T T T C A C A T.

3. LEVENSHTEIN DISTANCE TEST CASE 4: Population size: 700; number of generations: 110; crossover probability: 0.36; mutation probability: 0.03; size of each DNA sequence: 300; substring size: 24.

**RAT-HOUSE MOUSE RESULT:** Average time: 13 min 18 seconds; Distance achieved: 1; Closest substring: T A A A A A A G C A A A G C A C T G A A A A T G.

**RAT-FAT DORMOUSE RESULT:** Average time: 13 min 19 seconds; Distance achieved: 1; Closest substring: T A A A C G A A A G T T T G A C T A A G C T A G.

**RAT-COW RESULT:** Average time: 13 min 19 seconds; Distance achieved: 6; Closest substring: C A A A C A T C T A C C A C C C G G T T A A A A.

#### Observations

The expected result for this experiment should indicate that the rat is closer to the house mouse and fat dormouse, than the cow. We would also like to catch even a finer difference between the rat-house mouse distance and the rat-fat dormouse distance.

In the CSP setting, rank distance shows again a great difference between the rat-house mouse closest string, the rat-fat dormouse and the rat-cow closest string. Hamming is able to distinguish the rat from the cow genome, but it doesn’t catch the difference between the rat-house mouse closest string and the rat-fat dormouse closest string. The rat-fat dormouse Hamming distance appears to be smaller than the rat-house mouse Hamming distance, which is wrong. Levenshtein distance works as good as rank distance in this case, giving the expected result. Our observations are supported by the graphs shown in Figure 3.

In the CSSP setting, all distances perform very good and are able to put the rat genome near the house mouse and fat dormouse genomes rather than the cow genome. However, the rat-house mouse Hamming distance is very close to the rat-fat dormouse Hamming distance (the closest substrings differ only by one letter). The Levenshtein distance is the same for rat-house mouse and rat-fat dormouse closest substrings. The associated graphs are given in Figure 4.

In both CSP and CSSP settings, all distances are able to put the rat near the house mouse and fat dormouse rather than the cow, which is the expected result (see Figures 3 and 4). On top of this, RD is the only distance able to catch subtle differences, putting the rat DNA near the house mouse DNA rather than the fat dormouse DNA.

### Artificial Experiment

For this experiment we use only the CSSP setting. The goal of this experiment is to show the time improvement obtained by optimizing the genetic algorithm introduced in [29].

#### CSSP setting

1. TEST CASE 5: Population size: 500; number of generations: 100; crossover probability: 0.36; mutation probability: 0.02; size of DNA sequence 1∶90; size of DNA sequence 2∶90; substring size: 30.

DNA Sequence 1: A A A A A A A A A A A A T T T T T T T T T T T T T T T T T T T T T T T T T G G G G G A A A A A A A A A A A A A A A A A A A A A A A A A A A G G G G G G G G G G T T T T T A A A A A A A A A A A A A A A A.

DNA Sequence 2: C C C C C C C C C C G G G G G G G G G G T T T T T C C C C C C C C C C C C C C C C C C T T T T T T T T T T T T T T T T T T T T T T T T T G G G G G C C C C C C C C C C C C C C C C C.

**RANK DISTANCE RESULT:** Average time: 10 seconds; Distance achieved: 0; Closest substring: T T T T T T T T T T T T T T T T T T T T T T T T T G G G G G.

**HAMMING DISTANCE RESULT:** Average time: 35 seconds; Distance achieved: 0; Closest substring: T T T T T T T T T T T T T T T T T T T T T T T T T G G G G G.

**LEVENSHTEIN DISTANCE RESULT:** Average time: 3 min 22 seconds; Distance achieved: 0; Closest substring: T T T T T T T T T T T T T T T T T T T T T T T T T G G G G G.

#### Observations

Using an algorithm to compute rank distance in linear time and a hash table to store precomputed distances between DNA sequences, we are able to report a great improvement in terms of speed. The algorithm that computes rank distance in linear time was introduced in [14] and it takes advantage of the alphabet size (only four letters) to compute the distance. This algorithm doesn’t annotate the DNA strings, but it uses extra space to remember the position of each character in the DNA strings. We can reduce the time complexity of rank distance to be the same of Hamming distance using this linear time algorithm.

At the selection step, the genetic algorithm needs to sort the chromosomes in each generation by distance. In order to sort the chromosomes we must compare distances that are computed (or recomputed) between chromosomes and input sequences. Instead of computing the distances each time, we store the precomputed distances in a hash table. It is much faster to access a distance value stored in a hash table instead of computing it in linear time. Note that we also used the hash table optimization for Hamming and Levenshtein distances. This optimization helps us reduce the number of distances to be computed from 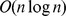 to 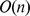.

For this test case, in [29] we reported a time of 58 minutes and 42 seconds. The average time in the same settings was reduced for 58 minutes to only 10 seconds. We recall that the average times are computed after running the algorithm 10 times on each test case using a computer with Intel Core i5 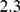 GHz processor and 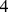 GB of RAM memory.

We obtained the same closest substring for each of the three metrics. This result shows that if an exact common substrings exists, the genetic algorithm can find it disregading the metric used. This shows that the genetic algorithm is robust and it can find the optimal solution if the input parameters are properly set.

### General Observations

We designed simple and clear experiments that can show the differences of the compared distances. In order to keep things simple, we used the genetic algorithms to determine the closest string or substring for only two DNA sequences. Of course, the algorithms work as well with multiple sequences at once, since the CSP and CSSP problems are generally defined for sets of strings.

We mention that the results obtained are not influenced by the fact that the DNA strings are part of coding or non-coding sequences or within genes or part of intergenic regions. The DNA strings used in our experiments were selected without taking into consideration these aspects so the strings may be part of any kind of region. However, it is important for DNA strings used in the same experiment to be extracted from the same position because the alignment matters. In other words, it doesn’t have sense to compare DNA from different regions that have different significance.

All our experiments show that RD can be computed 2 times faster than Hamming distance and 10 to 15 times faster than Levenshtein distance. As the closest string (or substring) size increases the Levenshtein distance takes more time to compute when compared to rank distance and Hamming distance.

Although the Hamming distance computes almost as fast as rank distance, the downside is that is gives inaccurate results compared to RD. The Levenshtein distance can easily be dismissed because is takes longer to compute and it is also unable to detect the subtle differences that rank distance detects by having a finer scale.

Neither Hamming distance or Levenshtein distance were able to give the right answer in all our experiments (Levenshtein distance is wrong in TEST CASE 1 and Hamming distance is wrong in TEST CASE 3). Only rank distance has the expected outcome in all the experiments. Overall, we believe that rank distance is best suited for finding closest strings or substrings on DNA sequences. Due to this observation we conducted the following experiment using only RD.

### Human-Chimpanzee-Gorilla Experiment

The goal of this experiment is to see if the DNA information can lead to one of the three distinct unrooted phylogenetic trees of higher primates. For this experiment we use only the CSP setting: we want to find the human-chimp closest string and the human-gorilla closest string and compare the associated rank distances. We perform four tests using DNA sequences of variable length and different input parameters for the genetic algorithm. We show graphs for the last three test cases which are more relevant.

#### CSP setting

In the first test case (TEST CASE 6) we use the first 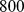 nucleotides extracted from each of the human, chimpanzee and gorilla DNA sequences. Obviously, the closest strings will also have 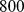 nucleotides.

The difference between the human, chimpanzee and gorilla mtDNA is very small and 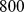 nucleotides may not be enough. Thus, for the next two test cases we use the first 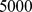 and 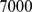 nucleotides (respectively) from the human, chimpanzee and gorilla to search for the closest string. In the last test case we will use 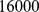 nucleotides (almost the entire DNA sequences).

We present only the distance achieved for each closest string, because the strings are too long to be presented here.

1. RANK DISTANCE TEST CASE 6: Population size: 7000; number of generations: 500; crossover probability: 0.36; mutation probability: 0.001; size of each DNA sequence: 800.

**HUMAN-CHIMPANZEE RESULT:** Average time: 7 min 3 seconds; Distance achieved: 43207.

**HUMAN-GORILLA RESULT:** Average time: 7 min 6 seconds; Distance achieved: 45544.

2. RANK DISTANCE TEST CASE 7: Population size: 33000; number of generations: 2000; crossover probability: 0.36; mutation probability: 0.0002; size of each DNA sequence: 5000.

**HUMAN-CHIMPANZEE RESULT:** Average time: 13–14 hours; Distance achieved: 426232;

**HUMAN-GORILLA RESULT:** Average time: 13–14 hours; Distance achieved: 358525.

3. RANK DISTANCE TEST CASE 8: Population size: 40000; number of generations: 2400; crossover probability: 0.36; mutation probability: 0.0001; size of each DNA sequence: 7000.

**HUMAN-CHIMPANZEE RESULT:** Average time: 27–28 hours; Distance achieved: 682664.

**HUMAN-GORILLA RESULT:** Average time: 27–28 hours; Distance achieved: 656806;

4. RANK DISTANCE TEST CASE 9: Population size: 55000; number of generations: 2800; crossover probability: 0.36; mutation probability: 0.00005; size of each DNA sequence: 16000.

**HUMAN-CHIMPANZEE RESULT:** Average time: 5 days and 6–7 hours; Distance achieved: 2412780.

**HUMAN-GORILLA RESULT:** Average time: 5 days and 6–7 hours; Distance achieved: 2089976.

#### Observations

We adjusted the genetic algorithm parameters to obtain the best results disregarding the higher computational time needed to get these results for the first three test cases. The graphs show that the size of the population used in the genetic algorithm is much higher than necessary because the best chromosome evolves very fast during the first 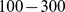 generations and then very slow. We also could of used less generations, but we wanted to make sure we catch every bit of information contained in the DNA.

In TEST CASE 9 the parameters are rather ajusted for speed than accuracy. We can obtain better approximations of the closest strings by using a population larger than 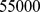 and a greater number of generations, but our results are more than good enough for this investigation.

The results for TEST CASE 6 shows that according to rank distance the human is near the chimpanzee rather than the gorilla. The graphs corresponding to TEST CASE 7, 8 and 9 from Figure 5 point to the other direction, that is the human is closer related to the gorilla.

It seems that 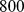 nucleotides are not enough to obtain a conclusive result. But our results are consistent when the length of the DNA is high enough. Here we show that using 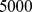 or 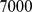 nucleotides we obtain that the human’s closest relative is the gorilla. We mention that we tested with more sequences of variable length and we obtained the same result for everything above 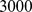 nucleotides to 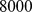 nucleotides. These results are consistent with our latest test case that uses 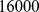 nucleotides.

Note that in our last test case we used almost all of the entire mtDNA which is approximately 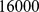 nucleotides long. We believe that the length of the DNA strings used in our experiment is enough to let us make a conclusion.

Overall, the DNA information that RD was able to extract during this experiment seems to support the theory favours the phylogenetic tree that joins the human with the gorilla [30]. However, we think more investigations in this area are needed to bring a strong conclusion to one way or the other.

### Conclusion and Further Work

In this paper we presented two genetic algorithms designed for solving the closest string problem and closest substring problem, respectively. The genetic operations for the closest string problem have a strong mathematical background and are only inspired from nature. The genetic algorithm designed for the closest substring problem uses standard genetic operations.

We tested these two algorithms using several experiments that involve DNA sequences extracted from mammals genomes. Each of these experiments were performed using three different metrics: rank distance, Hamming distance and Levenshtein distance. In all our experiments rank distance clearly outperforms Hamming and Levenshtein distances. On top of this, rank distance is the only distance able to catch subtle differences between DNA strings.

By comparing the results for each distance measure, we are able to conclude that RD is best suited for finding closest strings or substrings on DNA sequences.

We used our genetic algorithm with rank distance to bring some light in a case disputed by biology scientists: which is the closest human relative, the chimpanzee or the gorilla? The DNA information extracted by rank distance supports the theory that says the human closest relative is the gorilla. We also showed the importance of using DNA sequences that are long enough to obtain conclusive results. Too short DNA sequences can lead to confusing results.

In the near future we would like to compare our genetic algorithms based on RD with other approaches, such as dynamic programming techniques. We strongly believe that our approach is comparable, in terms of precision and speed, with other approaches.

We also want to investigate a possible approach to obtain better results. This approach combines the results coming from several parallel executions of the genetic algorithm. The best candidates from these parallel executions may be taken to form the first generation of another genetic algorithm. The best candidates will evolve together until the final result is achieved. The final result is expected to be an optimal solution. This approach could work very good with very high-dimensional input data.

## Methods

Genetic algorithms are adaptive searching techniques based on the principles of genetics (see [31] or [32]). The first genetic algorithms were introduced in [33] and [34]. The idea behind a genetic algorithm is to simulate the biological process of natural selection. A genetic algorithm applies a set of operations on a population over a number of generations. The population is a set of individual elements (called chromosomes) usually represented as binary strings. The set of operations applied on the population are inspired from biology: crossover (also called recombination), mutation, selection, etc. Genetic algorithms are used to solve optimization or search problems. A close-to-optimal solution should be enough when one wishes to use a genetic algorithm to solve a certain problem. In other words, one should not expect to get the optimal solution each time a genetic algorithm is executed.

### The General Algorithm

We used the classic general form of the genetic algorithm. For each problem, we used a different set of operations. The set of operations used for the closest substring problem are classical. The crossover and the mutation operations are the same operations found in nature. For the closest string problem the operations are only inspired from biology, but they rely on a mathematical background. We will later describe the structure of the chromosomes and the operations applied on each generation.

**Algorithm 1** *General Form.*

1: **Initialization**: Generate a random population that represents the first generation.

2: **Loop**: For a number of generations apply the next operations:

2.a 1em Apply the crossover according to the probability of having a crossover.

2.b 1em Apply mutations according to the probability of having a mutation.

2.c 1em Select the best candidates for the next generation using a density of probability.

3: **Termination**: Choose the best individual from the last generation to be the optimal ranking.

### The Closest String Problem Via Rank Distance (CSRD)

#### The chromosome

An individual chromosome is a permutation of ranks. Each chromosome is a possible candidate for the optimal ranking. Table 1 contains an example of three random chromosomes of length 9. Note that this is only an internal representation of the chromosomes. They are actually strings or DNA sequences.

We need to convert each input DNA to a permutation. Note that any string can be converted to a permutation. Each letter of the string can be annotated with an index that starts at 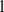 for each letter. For example, if we annotate the string “alibaba”, we will obtain “
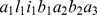
”. Now each letter is unique and can be replaced with a unique number. This is how we obtain the permutation. To obtain a string from the output permutation we only need a mapping from annotated letters to numbers. The mapping should be generated when the input strings are previously converted to permutations.

#### The crossover operation

There are three forms of crossover that are used by the algorithm. Each time the crossover must occur we apply all three forms of crossover.

The first crossover operation keeps the first part (prefix) of the individuals and completes the rest of the permutation according to the order given by the complementary chromosome. Table 2 gives the result of this crossover operation applied on chromosomes C1 and C2.

The second operation uses the same principle, but applies it at the other end of the chromosomes. This crossover operation keeps the last part (suffix) of the individuals and completes the rest of the permutation according to the order given by the complementary chromosome.

The third crossover is a natural combination of the previous two. This crossover keeps both the prefix and the suffix of the chromosomes but completes the middle part according to the order found in the complementary chromosome.

In order to successfully apply the crossover operations a certain cutting point should be randomly generated. There are six new individuals after the recombination because each crossover operation generates two new individuals. The best two individuals are chosen to replace the parent chromosomes. The optimality condition is used as a criterion to choose the best individuals.

We have chosen this model (with 3 types of crossover) because the use of a single crossover usually destroys certain parts of the two individuals involved in the operation. For example, the crossover that keeps the prefixes will have to reorder the components of the suffix. If this single type of crossover is used, we would be unable to evolve the suffix part of the chromosome. This will generate populations with similar individuals that tend to have a bad pattern. In this pattern a good part and a bad part always appear. With our model we ensure that individuals do not follow this pattern and get close to the optimal ranking, but in different ways.

#### The mutation

The mutation operation may be applied to any chromosome. The mutation only needs one chromosome. To apply a mutation on an individual two positions are randomly chosen. The values at the two positions are swaped.

Table 3 shows chromosomes C1, C2 and C3 with mutations. Although mutations are rare, multiple mutations may appear at the same chromosome. This situation is very unlikely.

#### The selection

To select the individuals for the new generation from the current generation we use a density of probability function. The new generation is involved in the next iteration of the algorithm. The first step is to sort the individuals on the maximal distances from the input rankings criterion in descending order. Then we generate indexes from the top to the bottom of the list of candidates. The indexes close to the top of the list are more probable. Note that one index can be generated several times; this is the case with the best candidates. There are also indexes that may never be generated; this is the case of the candidates close to the bottom of the list. The density probability function used to select the candidates for the next generation is the normal distribution of mean 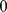 and variance 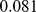 on the interval 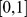:
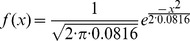



The graph of this function is represented in Figure 6.

Note that in the implementation of the algorithm the fitness function was statistically approximated.

The motivation for using this fitness function is based on test results. This fitness functions reduces the number of generations that are required to obtain a close-to-optimal solution. Helped by the crossover and mutation operations, the fitness function has a good generalization capacity: it doesn’t favour certain chromosomes which could narrow the solution space and lead to local minima solutions.

### The Closest Substring Problem via Rank Distance

#### The chromosome

Each chromosome is a sequence of DNA of fixed length that represents a possible candidate for the closest substring. Note that a sequence of DNA is simply a strand of nucleotides (A, C, G or T) that appear randomly in a sequence.

#### The crossover operation

The crossover operation between two chromosomes for the closest substring problem is straightforward. First, we need to generate a random cutting point. The prefixes of the two chromosomes remain in place, while the suffixes of the two chromosomes interchange. This is the standard crossover operation inspired directly from nature.

#### The mutation

To apply a mutation to a certain chromosome, one position is randomly chosen. The nucleotide found at that position will be changed with a new one. Multiple mutations may appear at the same chromosome, although this is very unlikely. This is the classic mutation operation that can also be found in nature.

#### The selection

The selection operation used here is similar to the selection used for closest string problem and is based on the normal distribution of mean 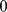 and variance 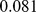 on the interval 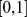. We have also tried other density probability functions such as 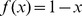, which has a better generalization capacity (it keeps a better variety of possible solutions over a greater number of generations). In our experiments we decided to go with the same function used for CSRD which makes the population evolve much faster. The lower generalization capacity can be compensated by increasing the size of the population.
